# A Case of Macrophage Activation Syndrome With Elderly Onset Still’s Disease Under Tocilizumab Treatment

**DOI:** 10.7759/cureus.59285

**Published:** 2024-04-29

**Authors:** Yuhei Fujisawa, Shigeto Horita, Keiko Wakabayashi

**Affiliations:** 1 Internal Medicine, Saiseikai Kanazawa Hospital, Kanazawa, JPN

**Keywords:** skin rash, fever, arthralgia, tocilizumab, macrophage activation syndrome (mas), adult-onset still’s disease

## Abstract

Adult-onset Still's disease in older adults is referred to as elderly onset Still's disease (EOSD). Few cases of tocilizumab (TCZ) use for EOSD management have been reported. Here, we report the case of an 87-year-old Japanese woman with EOSD who was not previously taking any medication. She had fatigue, sore throat, and loss of appetite for several days and gradually experienced difficulty walking. On examination, she was found to have a fever and erythema on the buttocks and extremities. Laboratory tests revealed leukocytosis with neutrophil predominance, elevated C-reactive protein (CRP) levels, and hyperferritinemia. A contrast-enhanced computed tomography scan of the chest to the abdomen showed no abnormalities. Antimicrobial therapy was initiated; however, the fever did not resolve. On day seven, 40 mg/day prednisolone (PDN) was started for EOSD in the absence of an obvious infection or a malignancy. On day 20, the fever recurred, and the patient was started on intravenous methylprednisolone (mPDN) half-pulse therapy (500 mg/day for three days). The fever resolved, and the CRP level decreased to 1 mg/dL but did not return to normal. On day 35, the fever recurred; therefore, 320 mg of TCZ was injected intravenously, and the PDN was tapered. On day 43, the patient tested positive for cytomegalovirus (CMV) antigenemia and improved on ganciclovir. On day 70, the patient developed fever, decreased white blood cell (WBC) and hemoglobin (Hb) levels, high lactate dehydrogenase (LDH) levels, hyperferritinemia, and elevated liver enzymes. Macrophage activation syndrome (MAS) was diagnosed due to hemophagocytosis on bone marrow examination. The patient was started on pulse therapy with glucocorticosteroids and cyclosporine. The patient’s fever decreased, and her WBC count and LDH level normalized. The patient continued rehabilitation for muscle weakness due to prolonged hospitalization and high-dose steroid use and was discharged from the hospital on day 150. The findings in this case suggest that the use of TCZ during the remission induction phase of EOSD may lead to MAS.

## Introduction

Adult-onset Still's disease (AOSD) is an autoinflammatory disease characterized by fever, arthralgia, and skin rash. The average age at the onset of AOSD in Japan is 40­-50 years [1]. Recently, some researchers have reviewed the clinical features of elderly onset Still's disease (EOSD) [2,3]. Tocilizumab (TCZ) has been used to manage EOSD in a few patients [4]. Moreover, only a few cases of macrophage activation syndrome (MAS) after EOSD treatment have been reported [5]. Herein, we report the case of a patient who presented with EOSD complicated by MAS after treatment with TCZ. This case was previously presented as a meeting abstract at the 67th Annual Scientific Meeting of the Japan College of Rheumatology on April 24-26, 2023.

## Case presentation

**Figure 1:**
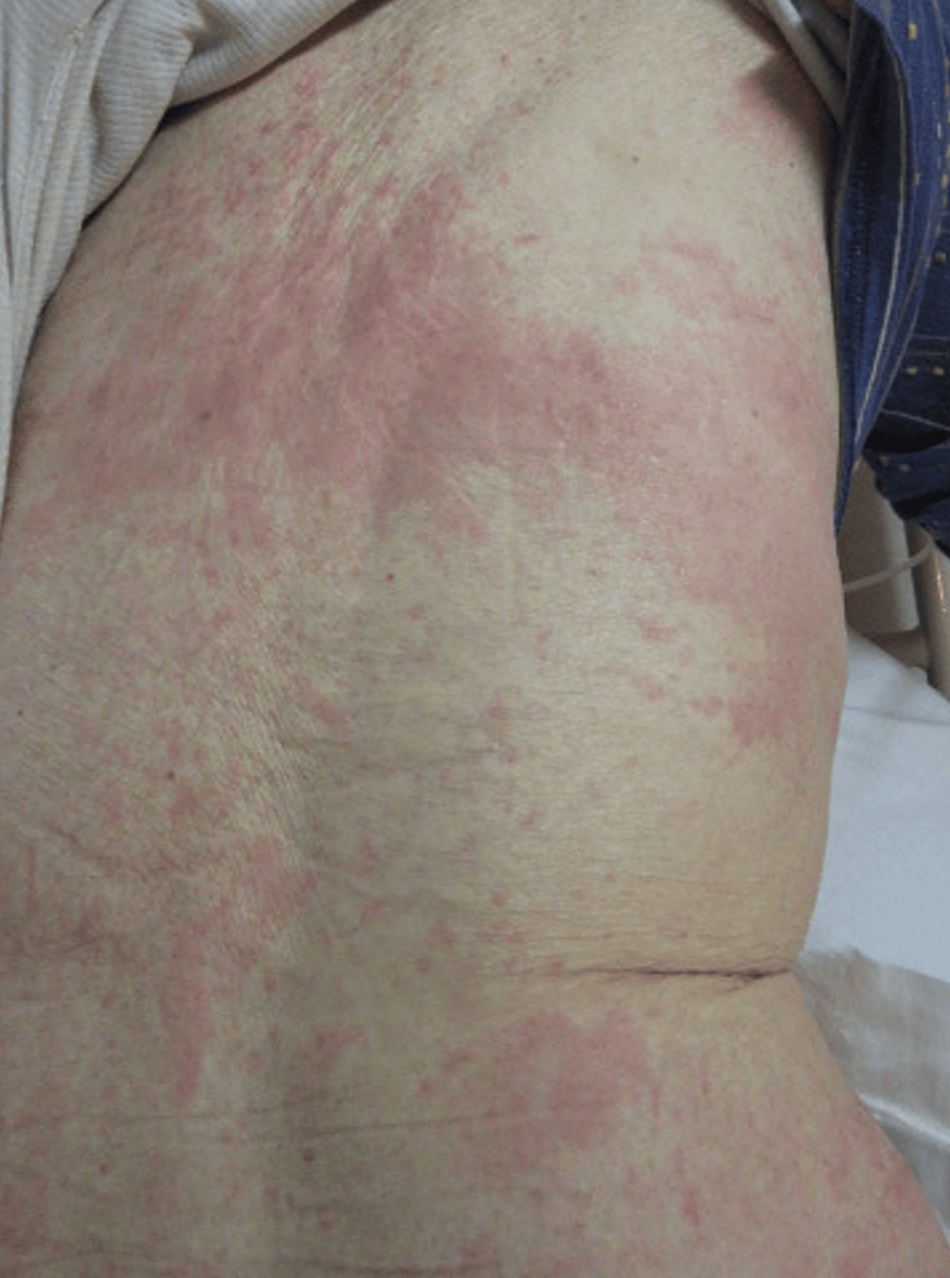
Skin findings. The patient presented with diffuse erythema, which partially coalesced on the buttocks and back and was more pronounced with fever.

An 87-year-old Japanese woman with persistent fever and sore throat for one week was admitted to our hospital. Her medical history was unremarkable, and she was not taking any medication. She had no recent travel or infections in the last two weeks. Furthermore, her family history was not indicative of rheumatic disease. Her vital signs on admission were as follows: temperature, 38.7℃; blood pressure, 147/88 mmHg; heart rate, 95 beats/min; and oxygen saturation, 95% in room air. Physical examination revealed erythema without tenderness or pruritus on the buttocks and back, which was exacerbated as the fever increased (Figure 1). She had no cervical or inguinal lymphadenopathy, no joint swelling, and no purpura. The laboratory tests revealed leukocytosis with neutrophil predominance and elevated levels of serum C-reactive protein (CRP), liver enzymes, lactate dehydrogenase (LDH), and ferritin (Table 1). She also tested positive for antinuclear antibodies and preexisting Epstein-Barr virus and cytomegalovirus (CMV) infections and negative for rheumatoid factors, antineutrophil cytoplasmic antibodies, and parvovirus B-19 immunoglobin M. Contrast-enhanced computed tomography of the chest to abdomen revealed no lymph node involvement or hepatosplenomegaly. Upper gastrointestinal endoscopy revealed no malignancy, and the stool human hemoglobin (Hb) test result was negative. After excluding malignancy and infection, AOSD was diagnosed based on the criteria of Yamaguchi et al. (three major points and two minor points) [6].

**Table 1:** Laboratory test data of the patient at admission and time of MAS development. MAS: macrophage activation syndrome; β2MG: β₂ microglobulin; WBCs, white blood cells; Lym: lymphocytes; Hb: hemoglobin; Plt: platelets; ESR: erythrocyte sedimentation rate; TP: total protein; Alb: albumin; AST: aspartate aminotransferase; ALT: alanine aminotransferase; LDH: lactate dehydrogenase; Na: sodium; K: potassium; Cl: chloride; BUN: blood urea nitrogen; Cr: creatinine; eGFR: estimated glomerular filtration rate; HbA1c: glycosylated hemoglobin; BNP: brain natriuretic peptide; FT4: free thyroxine; TSH: thyroid-stimulating hormone; Fe: iron; TIBC: total iron-binding capacity; CRP: C-reactive protein; IgG: immunoglobulin G; IgA: immunoglobulin A; IgM: immunoglobulin M; C3: complement component 3; C4: complement component 4; CH50: 50% hemolytic complement; ANA: antinuclear antibody; RF: rheumatoid factor; anti-CCP: anti-cyclic citrullinated peptide; MPO-ANCA: myeloperoxidase-antineutrophil cytoplasmic antibodies; PR3-ANCA: proteinase 3-antineutrophil cytoplasmic antibodies; sIL-2R: soluble interleukin-2 receptor; IL-6: interleukin-6

Urinalysis	On admission	Complicated by MAS		Normal range
Protein	1+	1+		
Occult blood	-	1+		
β2MG	45,682	123,396	μg/L	0-289
Blood cell count				
WBC	11,190	1,900	/μL	3,500-9,000
Neutrophil	90	42	%	42.0-74.0
Lym	8	39	%	18.0-50.0
Hb	9.5	9.2	g/dL	11.2-15.2
Plt	23.1	14.3	×10^4^/μL	14.0-37.9
ESR	50	15	mm/hr	
Blood chemistry				
TP	6.3	5.3	g/dL	6.5-8.2
Alb	2.0	2.6	g/dL	3.7-5.5
AST	100	141	U/L	10-40
ALT	33	77	U/L	5-45
LDH	693	867	U/L	120-245
Na	137	126	mEq/L	135-145
K	3.2	4.8	mEq/L	3.5-5.0
Cl	100	96	mEq/L	98-108
BUN	19.4	15.9	mg/dL	8.0-20.0
Cr	0.7	0.5	mg/dL	0.46-0.82
eGFR	59	82	ml/min/1.73m^2^	
HbA1c	6.2	-	%	4.6-6.2
BNP	74	-	pg/mL	0.0-18.4
FT4	1.47	-	ng/dL	0.75-1.45
TSH	0.37	-	μIU/mL	0.61-4.23
Fe	20	-	μg/dL	50-170
TIBC	162	-	μg/dL	250-460
Ferritin	14,448	5,973	ng/dL	5-157
D-dimer	5.0	-	μg/mL	<1.0
Serological test				
CRP	13.1	0.2	mg/dL	0.00-0.30
IgG	1397	-	mg/dL	820-1,740
IgA	296	-	mg/dL	90-400
IgM	92	-	mg/dL	52-270
C3	113	-	mg/dL	80-140
C4	26.9	-	mg/dL	11.0-34.0
CH50	55	-	U/mL	30-45
ANA	80 (Ho)	-	×	-
RF	3	-	U/mL	<15
anti-CCP antibody	<0.5	-	U/mL	<4.5
MPO-ANCA	<0.5	-	U/L	-
PR3-ANCA	<0.5	-	U/L	-
sIL-2R	2,617	3,542	U/mL	122-496
IL-6	18	68	pg/mL	<7.0

**Figure 2:**
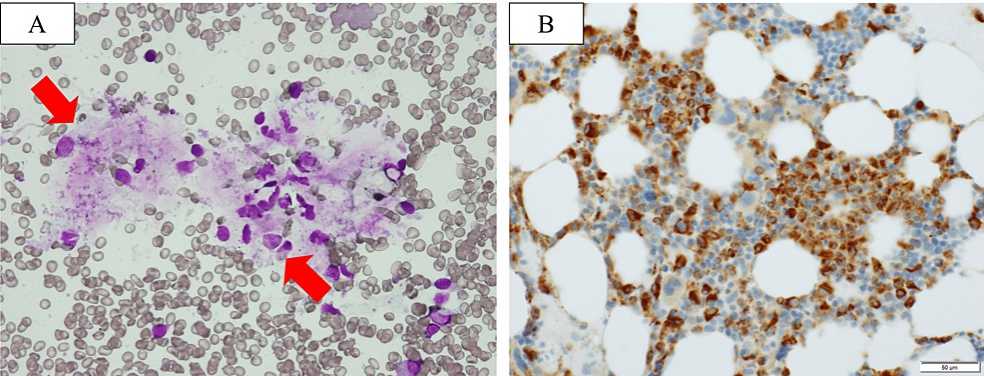
Bone marrow biopsy. A. May-Giemsa staining showing the phagocytosis of erythrocytes by macrophages (×100, red arrow). B. CD68-positive cells were observed at 100× magnification.

**Figure 3:**
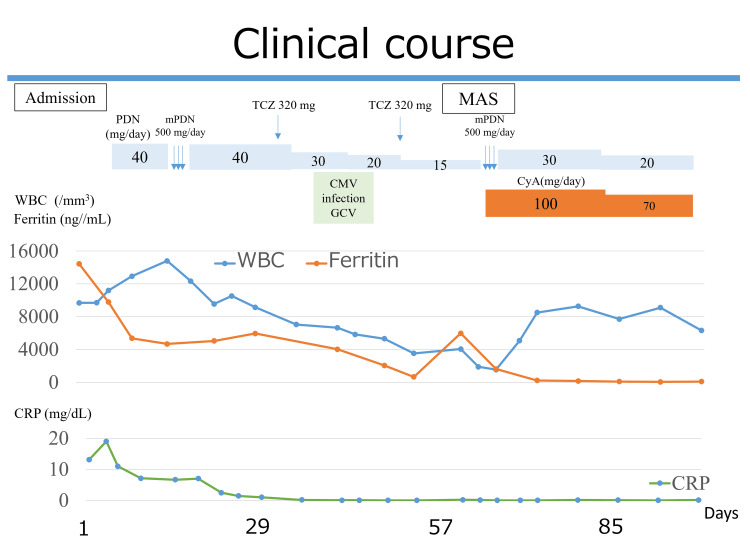
Clinical course during hospitalization. CMV, cytomegalovirus; CyA, cyclosporine; GCV, ganciclovir; MAS, macrophage activation syndrome; mPDN, methylprednisolone; PDN, prednisolone; TCZ, tocilizumab; WBCs, white blood cells

On the sixth hospital day, 40 mg/day prednisolone (PDN) was administered. However, she developed a fever and had elevated serum CRP and ferritin levels on the 21st day of hospitalization. Therefore, intravenous methylprednisolone (mPDN) half-pulse therapy (500 mg/day for three days) was administered, and her symptoms improved the following day. Her serum CRP and ferritin levels also decreased. Starting on the 35th hospital day, TCZ (320 mg by intravenous injection) was administered every two weeks. A total of two doses were administered. Thereafter, her serum CRP levels normalized. On the 40th day of hospitalization, cytomegalovirus reactivation was diagnosed because the patient was positive for cytomegalovirus antigenemia. She was treated with intravenous ganciclovir. On the 70th hospital day, she had a fever, decreased appetite, and fatigue. Laboratory test findings revealed decreased white blood cell (WBC) and Hb levels and increased serum LDH, ferritin, and liver enzyme levels (Table 1). Bone marrow biopsy findings confirmed the presence of hemophagocytosis without any signs of malignancy (Figure 2), resulting in a diagnosis of MAS. Intravenous mPDN half-pulse therapy with cyclosporine was administered, resulting in an immediate improvement in the patient’s symptoms and normalization of WBC, Hb, and LDH levels. She was discharged on the 150th hospital day after long-term rehabilitation because of her poor general condition and muscle weakness (Figure 3). The patient has remained in remission with normal serum CRP and ferritin levels for two years. The Naranjo scale can reach three points [7].

## Discussion

We describe a patient with EOSD who was treated with high-dose PDN. However, TCZ was administered to induce remission because CRP levels did not decrease, and the patient developed a high fever. Two weeks after the second dose of TCZ, the patient developed MAS. Thereafter, TCZ was discontinued, and high-dose PDN and cyclosporine were initiated; no relapse was observed over the next two years.

Several clinical studies on EOSD have been conducted. A review of 40 patients with EOSD revealed the use of TCZ in four patients with EOSD, including one patient in which TCZ was used after MAS onset [4]. Compared to younger patients, there are significantly more reports of older patients with EOSD with MAS and related complications, as well as a greater complication rate of CMV infection [2]. However, it should be noted that AOSD has been reported to complicate MAS, resulting in severe illness and death [8]. In contrast, no significant differences in MAS complications were observed between younger patients and those with EOSD [3,9]. Since the MAS complication rates associated with AOSD and EOSD differ between studies, additional data from case reports and multicenter studies are needed.

Several cases of MAS development after TCZ administration in patients with AOSD have been reported [5,10-13]. However, none of these reports have demonstrated a causal relationship between TCZ and the development of MAS in patients with AOSD. Although the exact mechanism underlying the development of MAS during anti-IL-6 treatment remains unclear, it is presumed that the selective inhibition of a subset of pathways affects other immune signaling pathways that induce macrophage hyperactivation. In contrast, the results of a physician-initiated clinical trial showed that TCZ use was beneficial in patients with treatment-resistant AOSD, and there have been no reports of MAS development related to TCZ use [14]. Our patient developed MAS after TCZ treatment during the remission induction period for EOSD. Based on this clinical course, we cannot exclude the possibility that PDN and TCZ did not suppress EOSD activity and that MAS developed. It is desirable to collect cases in which TCZ was introduced to induce remission in patients with EOSD to determine whether they developed MAS.

## Conclusions

We described a case in which MAS developed after TCZ treatment for EOSD. The results seem controversial because some patients develop MAS after TCZ use, while others do not. Therefore, the optimal timing of TCZ administration for treating EOSD and AOSD should be investigated in future studies.
